# Enhancement of corrosion resistance of the cooling systems in desalination plants by green inhibitor

**DOI:** 10.1038/s41598-020-61810-9

**Published:** 2020-03-16

**Authors:** M. A. Deyab, Eric Guibal

**Affiliations:** 1 Egyptian Petroleum Research Institute (EPRI), Nasr City, Cairo Egypt; 2 Polymers Composites and Hybrids, IMT – Mines Ales, 6, avenue de Clavières, F-30319 Alès cedex, France

**Keywords:** Green chemistry, Materials chemistry

## Abstract

*Taraxacum officinale* extract (TOE) has been tested for preventing the corrosion of cooling systems in desalination plants. The inhibition of corrosion effects has been characterized by chemical and electrochemical methods (Mass loss, potentiodynamic polarization and electrochemical impedance spectroscopy) and surface observations. Tests on cooling systems were carried out in seawater environment. The presence of TOE in the re-circulation loop decreases the corrosion of carbon steel by adsorption of TOE compounds on the surface of metal pipes. The optimum TOE concentration was reached at 400 mg L^−1^ and the inhibition efficiency was higher than 94%. TOE allowed increasing the energy barrier of the corrosion process. SEM, FT-IR and UV spectra observations confirmed that TOE prevents corrosion attacks at the surface of the pipes. HPLC analyses identified the presence of saccharides, organic acids, phenol antioxidant and caffeic acid derivatives in TOE, which may be the active promoters of corrosion inhibition.

## Introduction

Most of the equipment used in the design of desalination plants, such as cooling pipes, is built in carbon steel. Exposed to aggressive oxidizing conditions, such as seawater, these cooling systems (re-circulating pumps and pipes) are seriously damaged by corrosion^1–4^. This is a worldwide problem with crucial importance in arid areas where desalination is a critical process for water supply.

The prevention of corrosion effects is usually operated using inhibitors to reinforce the physico-chemical stability of equipment and to limits the damaging effects of cooling fluids on pipes. Corrosion inhibitors are generally added to cooling solutions to inhibit the metal corrosion and reduce the aggressive action of cooling solutions^1,5–7^. Recently, the use of synthetic chemical compounds as corrosion inhibitors was restricted because of their possible toxic effects^8^. Alternative bio-sourced corrosion inhibitors have recently received a great attention from research community because of the abundance and low toxicity of these natural products. These natural corrosion inhibitors are mainly extracted from plants^9–20^ and seaweed biomass^21–23^.

In this work, a new bio-sourced extract has been investigated for the first time for preventing corrosion of carbon steel in aggressive environment (desalination plant conditions). This extract (TOE) was obtained from *Taraxacum officinale*, which is a compound used in Chinese medicine and in various modern pharmaceutical formulations^24,25^. *Taraxacum officinale* is well known for its antioxidant activity, having a high content of polyphenols such as sesquiterpene, lactones, phenylpropanoids, triterpenoid saponins and polysaccharides^26,27^. Most of these compounds contain heteroatom (e.g., nitrogen and oxygen) and multiple bonds in their molecules; this may contribute to facilitate the adsorption of TOE at the surface of metal pipes.

This plant can be found worldwide and has been found in food artefacts since Prehistorical times. Therefore, TOE can be considered innocuous in terms of ecological risks.

TOE can be readily and inexpensively extracted from abundant and widely disseminated resources. The extraction of active portions of the plant is processed using selective solvent methods^25,27–29^. It is noteworthy that TOE is easily biodegradable.

These different properties make this extract a promising green candidate for inhibiting the corrosion of pipes by re-circulating highly saline cooling fluids. The current work investigates the possibility to substitute this natural inhibitor to chemical organic and inorganic formulations for promoting environmental sustainability in desalination plants.

The properties of corrosion inhibition have been characterized by SEM, UV and FT-IR observations of the surface of metal supports exposed to seawater solution in the presence of increasing concentrations of TOE. The effect of the inhibitor was also quantified by chemical and electrochemical methods. The effects of immersion time and temperatures on corrosion inhibition have been also investigated. In addition, the composition of TOE was analyzed by HPLC; in order to explain its anti-corrosion effect.

## Materials and Methods

### Materials

Hurgada Desalination Plants (Egypt) supplied, as sheets and rods, the carbon steel material tested in this study. The reported composition of the material obeys the following specifications: C = 0.06 wt.%; Mn = 0.005 wt.%; Si = 0.7 wt.%; S = 0.012 wt.%; P = 0.001 wt.%; Ni = 0.015 wt.%; Cr = 0.004 wt.%; Mo = 0.002 wt.% and Fe, mass balance to 100%). Prior to tests, the carbon steel material was abraded using a series of emery paper sheets (from 600 to 1200 grains) and finally washed with water and acetone.

*Taraxacum officinale* extract (TOE, 2:3 aqueous/ethanol solution) was purchased from Symrise (Holzminden, Germany); dry extract in the commercial product represents 50% (w/w). The addition of TOE in the cooling fluid was varied between 100 and 400 mg L^−1^ (equivalent dry extract mass). The analysis of the compounds in the TOE was carried out using a High Performance Liquid Chromatograph (HPLC 9030, Shimadzu, Kyoto Japan), equipped with a capillary column (Shim-pack VP-ODS; 4.6 m × 150 mm × 4.6 µm). The mobile phase was de-mineralized water (fed with a flow rate of 1.2 mL min^−1^), while the injection volume was 20 µL. The composition of seawater sample (collected at Ain Sokhna, Egypt) is appearing (major elements) in Table 1.

**Table 1:** Seawater composition (major elements).

Compound	Concentration (mg L^−1^)
Potassium	23677
Magnesium	14233
Bromide	433
Hydrogen carbonate	2543
Sodium	1654
Chloride	397
Sulfate	103
Calcium	95
Total dissolved solids (TDS)	44135

Concentrations were determined by Dionex™ ICS-6000 Capillary HPIC™ System - Thermo Fisher Scientific (Waltham, MA, USA).

### Evaluation of mass loss

Carbon steel sheets were cut into 2.7 cm × 1.6 cm × 0.05 cm pieces (surface area, *A*: 4.32 cm^2^) for evaluating mass loss measurements (the average weight of samples was 1.2543 g). The carbon steel sheets were immersed into 100 mL seawater sample or TOE-treated seawater. The mass loss was determined according to ASTM G 01 method^30^. The samples were immersed in seawater for 168 h (*t*). The experiments were performed in triplicate and the average mass loss (*W*, mg) was calculated. The corrosion rate (*C*_*R*_, mg cm^−2^ h^−1^) was determined according to:

1 $${C}_{R}=\frac{W}{A\times t}$$

The corrosion inhibition efficiency (*η*_*W*_%) was evaluated according to:

2 $${\eta }_{W} \% =\frac{{{C}}_{{\rm{R0}}}-{{C}}_{{\rm{R}}}}{{{C}}_{{\rm{R0}}}}\times 100$$

where, *C*_R0_ is the corrosion rate in absence of TOE (i.e., “natural” corrosion).

### Electrochemical analysis

The electrochemical characterizations were performed through electrochemical impedance spectroscopy (EIS) and potentiodynamic polarization using a potentiostat/galvanostat (Gill AC 947, ACM Instruments, Grange-Over-Sands, Cumbria, UK). The experimental set-up is constituted of a 3-electrode glass cell (equipped with a standard calomel electrode (SCE), a platinum foil and carbon steel rod, with exposed surface area = 0.458 cm^2^). The EIS system was processed in the 30 kHz-1.0 Hz frequency range, with an amplitude of 10 mV peak-to-peak, at open circuit potential (OCP). The polarization system was processed in the Tafel region with scan rate of 1.25 mV s^−1^.

A stabilization time of 30 min was applied before proceeding to electrochemical experiment. The inhibition efficiency (*η*_*R*_%) from EIS data is obtained by^31^:

3 $${\eta }_{R} \% =\frac{{{R}}_{{\rm{ct}}}-{{R}}_{{\rm{cto}}}}{{{R}}_{{\rm{ct}}}}\times 100$$

where *R*_ct_ and *R*_cto_ are the charge transfer resistances in the presence and absence of TOE, respectively.

The inhibition efficiency (*η*_j_%) from polarization data is obtained by^32^:

4 $${{\rm{\eta }}}_{j} \% =\frac{{{j}}_{{\rm{corr}}(0)}-{{j}}_{{\rm{corr}}}}{{{j}}_{{\rm{corr}}(0)}}\times 100$$

where *j*_corr_ and *j*_corr(0)_ are the corrosion current densities in the presence and absence of TOE, respectively.

### Surface morphology analysis

A scanning electron microscope (SEM, JEOL JEM-1200EX II, Jeol, Ltd, Tokyo, Japan) was used for the direct observation of the damages at the surface of carbon steel sheets after exposure of seawater for 168 h, in absence or presence of TOE. Small samples were cut from carbon steel sheets and their surface was cleaned with ethanol to remove any deposited corrosion product prior to SEM analysis. The samples were not sputter-coated.

UV and FT-IR spectra of the TOE-seawater before and after immersion of the carbon steel sample were conducted using Perkin–Elmer UV–visible L spectrophotometer and FTIR spectrophotometer (Shimadzu), respectively.

## Results and Discussion

### Chemical composition of TOE extract

**Figure 1:**
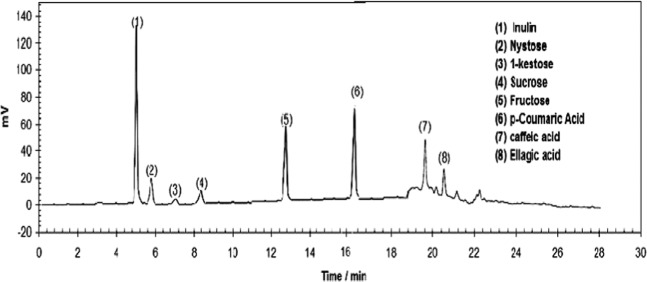
HLPC chromatogram of TOE (main compounds).

**Figure 2:**
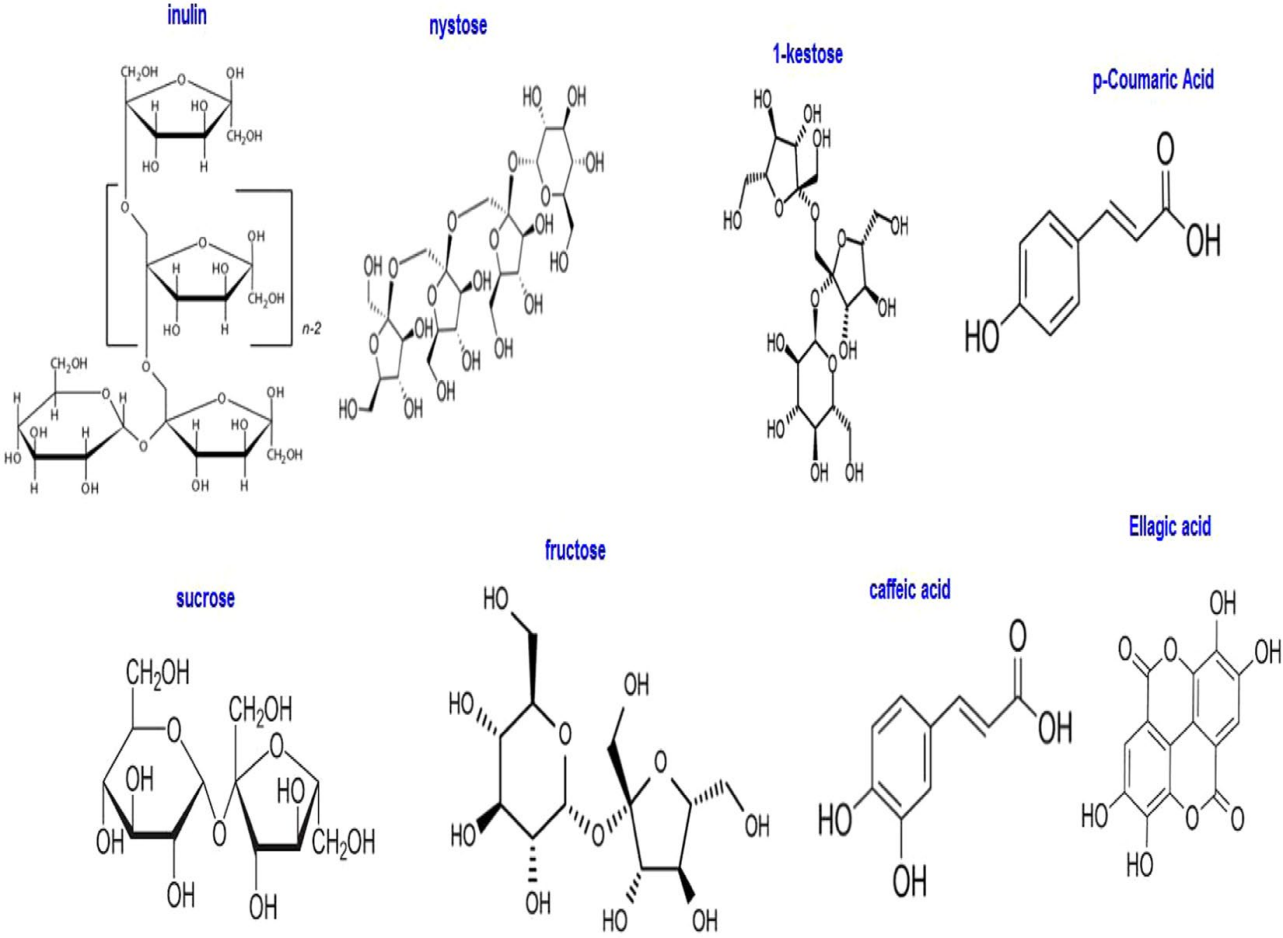
Molecular structures of main TOE compounds.

The TOE extract is predominantly composed of a few of organic compounds. Several studies have reported the composition of different parts of *Taraxacum officinale* plant for nutritional facts and presence of vitamins and active principles^25,27^. The extraction process obviously influences the composition of the solution: Jung *et al*.^33^ compared the ^1^H NMR profiles of aqueous and organic extracts (for another species of *Taraxacum* plant, *T. coreanum*); Kenny *et al*. also observed substantial differences in the extracted species while using aqueous or solvent extracting agents^29^. The aqueous extract is mainly constituted of saccharides such as glucose, sucrose; however, many other amino-acids, polyphenols^26^, flavonoids^25^ and organic acids^28^ are also identified. The current analysis of the TOE extract by HPLC shows the main compounds of the corrosion inhibitor (Fig. 1). The chromatogram confirms the presence of inulin, nystose, 1-kestose, sucrose and fructose (Figs. 1 and 2). These sugars (formed of monosaccharide and oligosaccharides) are natural reducing agents that can contribute to reduce the oxidizing conditions of metal immersion solution. It is noteworthy that *Taraxacum* extracts have, in addition to antioxidant properties, a natural antimicrobial activity^29^, which may contribute to minimize also bio-corrosion phenomena. This effect is usually associated to phenolic-based compounds contained in ethanolic extracts of the plant. At higher retention times, the chromatogram shows a series of peaks that are assigned to phenolic-based compounds in the water/alcohol extract: some of these peaks have been identified including p-coumaric acid, caffeic acid and ellagic acid. These are polyphenols that were already identified in TOE^26,27,29,33^.

### Mass loss study

**Figure 3:**
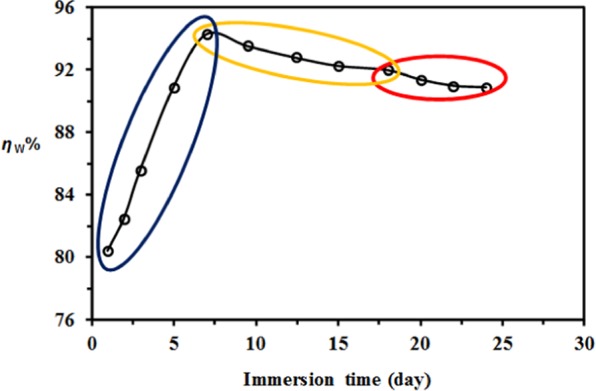
Effect of immersion time of carbon steel in seawater on inhibition efficiency (*η*_W_%) in the presence of TOE (mass loss method; Experimental conditions: dosage of TOE: 400 mg L^−1^, volume of solution: 100 ml, surface of steel sheet: 4.32 cm^2^, T: 298 K).

Figure 3 shows the effect of time on the inhibition efficiency (*η*_W_%). The efficiency of TOE increases with contact time up to 7 days (from 80 to 94%) and tends to slightly decrease at higher contact time; however, even at long contact time (i.e., 24 days) the inhibition efficiency remains close to 91%. This means that despite the possible degradation of the extract, the protecting effect is maintained over 21 days; the extract compounds kept adhering to carbon steel surface. For further experiments, the contact time was set to 7 days (i.e., 168 h) for evaluating corrosion inhibition performance.

The immersion of carbon steel sheets for 168 h in seawater allows evaluating the corrosion rate in function of the concentration of TOE through the variation of mass loss (Table 2). The corrosion rate exponentially decreases with the concentration of TOE:

5 $${C}_{R}=4.89\,{e}^{-0.0073TOE}\,({R}^{2}=0.990)$$

**Table 2:** Mass loss data for carbon steel immersed in seawater in the presence of increasing amounts of TOE at 298 K.

TOE concentration (mg L^−1^)	*C*_*R*_ (mg cm^−2^ h^−1^)	*η*_*W*_ (%)
0 - Blank	4.45	—
100	2.41	45.8
200	1.37	69.2
300	0.50	88.7
400	0.25	94.3

**Figure 4:**
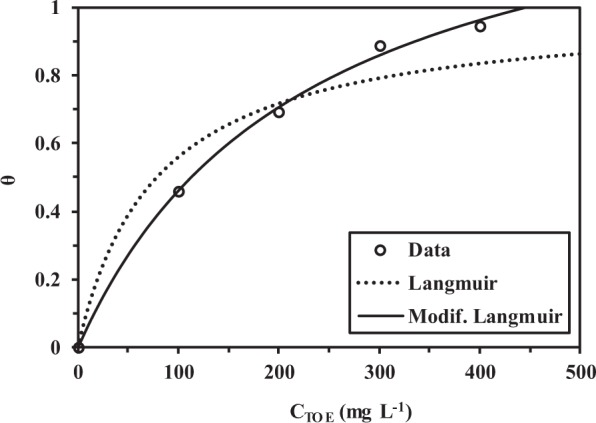
Modeling of sorption isotherm using the Langmuir (Eq. 7) and the modified Langmuir (Eq. 8) equations for corrosion rate in function of TOE concentration.

The corrosion inhibition efficiency (*η*_W_%) clearly reflects this trend: the efficiency of corrosion inhibition linearly increases with TOE concentration from 100 to 400 mg L^−1^ and but tends to stabilize above. At 400 mg L^−1^ dosage of inhibitor, the efficiency exceeds 94%. Pramudita *et al*.^14^ investigated the effect of tanning concentration (increased by increasing maceration time of *Terminalia catappa* leaves) on the corrosion rate of mild steel in 1 M sulfuric acid solution: the reduction of corrosion rate is not significant when the concentration of tannins exceeded 500 ppm. In the case of *Gingko* leaves extract, the limit concentration for stabilization of inhibition efficiency was around 50 mg L^−1^. For the extract of seeds of *Nigella sativa* L., the limit concentration for the stabilization plateau was close to 1 g L^−1^ ^34^. The surface coverage (*θ*, or fractional inhibition efficiency) can be defined by^35^:

6 $$\theta =\frac{{C}_{R0}-{C}_{R}}{{C}_{R0}}$$

which is correlated to inhibitor concentration (C, mg L^−1^) by the Langmuir-type sorption equation:

7 $$\theta =\frac{K\times C}{1+K\times C}$$

where K is the sorption equilibrium coefficient (L mg^−1^). As a first approximation (compared to conventional application of Langmuir equation) C concentration is considered unchanged by sorption on the surface (negligible variation). Figure 4 shows the variation of surface coverage as a function of TOE concentration. The equation roughly describes the sorption behavior of TOE on carbon steel sheets; and K value (determined by the non-linear regression fit of experimental data with Eq. 7; R^2^: 0.956) is close to 0.013 L mg^−1^. In the case of Gingko leaves extracts Deng *et al*.^35^ reported values in the range 0.17–0.57 L mg^−1^, depending on the type of acidic solution. In the case of *Silybum marianum* leaves extract, values of sorption constant were reported to vary between 0.017 and 0.063 L mg^−1^ ^36^. For *Aster koraiensis* leaves extract the sorption constant reaches 0.71 L mg^−1^ ^37^. Casein was also reported for inhibiting mild steel corrosion in acidic solutions with sorption constant ranging between 0.006 and 0.034 L mg^−1^ (depending on temperature)^38^. For the extracts of date palm leaves and seeds, the sorption constant also varied in a similar range (between 0.004 and 0.18 L mg^−1^)^39^. The comparison of these constants is made difficult by substantial differences in the experimental conditions; however, the sorption constant is roughly of the same order than most of reported data.

Recently, Samontha and Lugsanangarm^40^ used a derived form of the Langmuir equation adapted to corrosion systems:

8 $$\theta =\frac{K\times C}{n+n\times K\times C}$$

where n is a constant. Obviously, using a supplementary fitting parameter improves the quality of the fit; the determination coefficient increases up to 0.998. The sorption constant is close to 0.0044 L mg^−1^ while the n constant is close to 0.663.

### Thermodynamic aspects

The temperature slightly affects the corrosion rate. Table 3 shows that the corrosion rate (*C*_R_) increases with temperature between 298 K and 328 K (from 4.45 to 5.88 mg cm^−2^ h^−1^). In the presence of TOE (at the concentration of 400 mg L^−1^), the corrosion rate also increases but keeps below 0.53 mg cm^−2^ g^−1^. It is noteworthy that the inhibition efficiency (*η*_W_%) slightly decreases from 94.3 to 90.9%. This is probably associated to a partial desorption of TOE molecules from the surface of carbon steel sheet. Similar trends were reported for corrosion inhibition using seaweed extract^21^ and provitamin B5^41^. On the opposite hand, in the case of *Silybum marianum* leaves increasing the temperature improved the inhibition efficiency^36^; similar trend was reported by Rabizadeh *et al*. while using casein as the inhibitor^38^, and by Umoren *et al*.^39^ with palm seed ethanolic extract.

**Table 3:** Mass loss data at different temperatures for carbon steel immersed in seawater in the presence/absence of TOE (400 mg L^−1^).

Temperature (K)	TOE	*C*_*R*_ (mg cm^−2^ h^−1^)	*η*_*W*_ (%)
298	0	4.45	—
	+	0.25	94.3
308	0	4.84	—
	+	0.29	94.0
318	0	5.20	—
	+	0.37	92.8
328	0	5.88	—
	+	0.53	90.9

**Figure 5:**
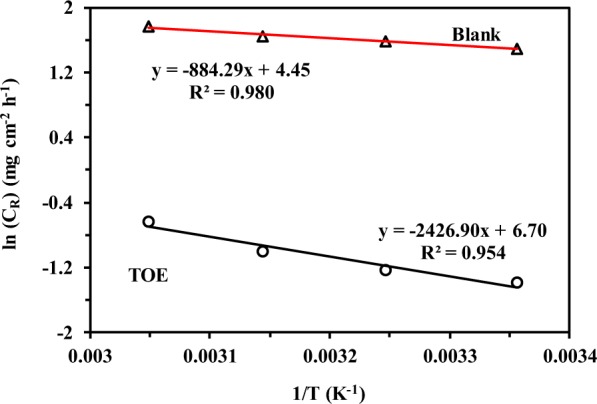
Effect of temperature on corrosion rates (*C*_R_, mg cm^−2^ h^−1^) for carbon steel sheet immersed in seawater – Comparison of Arrhenius plots for reference (blank, absence of TOE) and TOE-treated seawater (mass loss method; Experimental conditions: dosage of TOE: 400 mg L^−1^, volume of solution: 100 ml, surface of steel sheet: 4.32 cm^2^).

The Arrhenius plot was used for evaluating the activation energy (*E*_a_, kJ mol^−1^) of corrosion for carbon steel in seawater and TOE-treated seawater, using the equation^42^:

9 $${C}_{R}=A{e}^{\frac{-{E}_{a}}{RT}}$$

where *R* is the universal gas constant 8.314 J mol^−1^ K^−1^, *T* the absolute temperature and *A* the pre-exponential factor. Figure 5 compares the Arrhenius plots for the two systems. The activation energy increases with the presence of TOE: from 7.34 kJ mol^−1^ to 20.16 kJ mol^−1^. High activation energy is necessary for retarding the corrosion of carbon steel in the presence of TOE^43^. The adsorption of TOE at the surface of samples leads to an increase of the double layer thickness, which in turn, increases the energy barrier required for initiating the corrosion process^44^. This behavior was attributed to a preferential physical sorption of TOE compounds (over chemical sorption mechanism)^45^. Rabizadeh and Asl^38^ transformed the Arrhenius equation to determine the thermodynamic activation parameters (entropy change, Δ*S*, and enthalpy change Δ*H*, equivalent to activation energy) according to:

10a $${C}_{R}=A{e}^{\frac{-{E}_{a}}{RT}}=\left(\frac{RT}{Nh}{e}^{\frac{\Delta S}{R}}\right)\times {e}^{\frac{-\Delta H}{RT}}$$

10b $$\mathrm{ln}\left[\frac{{C}_{R}}{T}\right]=\left[\mathrm{ln}\left(\frac{R}{Nh}\right)+\left(\frac{\Delta S}{R}\right)\right]-\frac{\Delta H}{R}\times \frac{I}{T}$$

where *N* is the Avogadro constant (6.2022 10^23^ mol^−1^) and *h* is the Planck constant (6.6261 10^−34^ m^2^ kg s^−1^).

The relation ln(C_R_/*T*) vs. 1/*T* in Eq. 10b was drawn in the linear form (this figure is not presented here). The values of Δ*H* can be calculated from the slopes of the straight lines in the relation ln(C_R_/*T*) vs. 1/*T*. The slopes of the straight lines in the absence and presence of TOE are 571 and 2114, respectively. From the above calculations, The Δ*H* value increases from 4.75 kJ mol^−1^ to 17.58 kJ mol^−1^ in the presence of TOE. Similar drastic increase in enthalpy change was observed by Rabizadeh and Asl^38^ when using casein as an inhibitor of corrosion for mild steel. The positive value of the enthalpy change is associated with the endothermic character of the steel dissolution^38^. The entropy change (deduced from the intercept of ln(C_R_/*T*) vs. 1/*T*) slightly varied from −235 J mol^−1^ K^−1^ to −216 J mol^−1^ K^−1^. Similar variations were observed in the case of *Centaurea cyanus* (aqueous cornflower) extract^41^, *Houttuynia cordata* extract^46^ or *Sapindus* extract^47^. While comparing the entropy changes in presence and absence of coffee husk extract, Cordeiro *et al*.^48^ obtained very close values and they concluded that the mechanism was a simple substitution of water sorbed at the surface of metal shift with the molecules present in the extract. In the present case, the differences between the entropy changes for seawater and treated seawater are more important; this means that an additional mechanism is probably involved in the process.

The change in Gibbs energy (Δ*G*, kJ mol^−1^) can be deduced at 298 K by Eq. 11:

11 $$\Delta G=\Delta H-T\Delta S$$

The free Gibbs energy (for corrosion) increases with addition of TOE in seawater from 74.8 to 82.0 kJ mol^−1^. The positive value of Δ*G* means that the reaction is not spontaneous and that the corrosion requires higher energy in the presence of TOE.

### Electrochemical measurements

**Figure 6:**
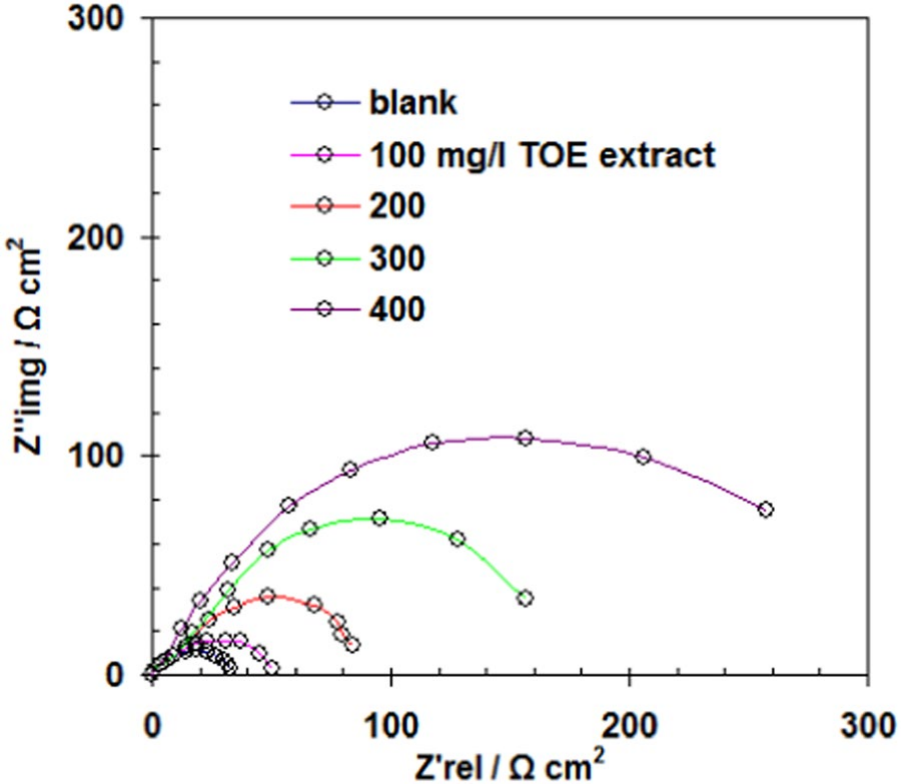
Nyquist plot for carbon steel immersed in seawater - Comparison between raw seawater and seawater treated with increasing amounts of TOE (Experimental conditions: volume of solution: 100 ml, surface of steel sheet: 0.458 cm^2^, T: 298 K).

**Figure 7:**
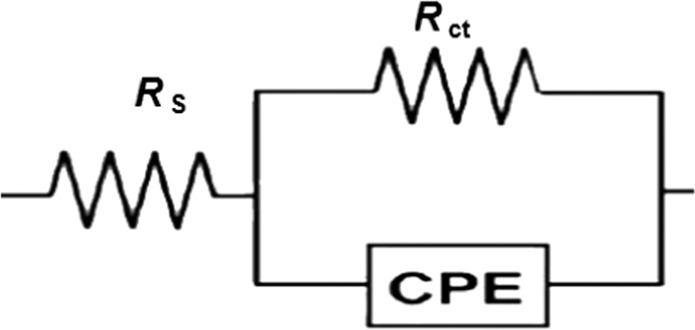
Schematization of electrochemical system.

Electrochemical impedance spectroscopy was applied to samples exposed to seawater for 30 min at T: 298 K. Figure 6 shows the Nyquist plots for increasing concentrations of TOE. The size of semi-circles progressively increases with the amount of TOE. This is usually associated with charge transfer mechanism^7,49,50^, and with an increase of the resistivity of carbon steel surface. Figure 7 schematizes the equivalent circuit that includes a charge transfer resistance (*R*_ct_), a constant phase element (CPE) and solution resistance (*R*_s_). In the case of fucoidan coating for the inhibition of zinc corrosion in seawater Wang *et al*.^51^ reported a typical double semicircles for Nyquist plots; this mechanism was associated to two in-chain mechanisms of resistance to charge transfer and to film resistance (diffusion mechanism) and two capacitances (double-layer and membrane capacitances). Chellouli *et al*.^34^ also reported irregular semicircles when using *Nigella sativa* L.

The values of the different parameters of EIS (*R*_ct_, *R*_s_ and CPE) are reported together with the inhibition efficiency (*η*_R_%) in the Table 4. Increasing the concentration of TOE drastically increases the charge transfer resistance (by a factor close to 11) while the CPE strongly decreases (from 5.2 to 0.3 µF cm^−2^). This strong decrease is explained by the increase in the thickness of the electrical double layer (due to the surface sorption of TOE extract compounds)^52,53^ and/or the decrease in local dielectric constant^17,48,54,55^.

**Table 4:** Impedance parameters and inhibition efficiency for carbon steel in seawater in the presence of increasing concentrations of TOE at 298 K.

TOE concentration (mg L^−1^)	*R*_s_ (Ω cm^2^)	*R*_ct_ (Ω cm^2^)	*CPE* (µF cm^−2^)	*η*_R_ (%)
0 - Blank	0.67	33.8	5.2	—
100	0.69	58.4	4.1	42.1
200	0.76	90.3	1.2	62.5
300	0.86	160.2	0.5	78.9
400	0.89	355.3	0.3	90.4

However, at TOE concentration higher than 200 mg L^−1^, the decrease tends to stabilize. Similar behavior was observed for corrosion inhibition using berberine extracted from *Coptis chinensis*^56^, berberine dye^57^, spices extract (curcuma, safran)^17^, acacia leaves (*Acacia cyanophylla*) extract^58^, irbesartan^43^. In the case of coffee husk extract the variations in the CPE were much less marked^48^.

On the other hand, the resistance to charge transfer (*R*_ct_) linearly increases with TOE concentration below 200 mg L^−1^, and exponentially above this limit concentration. However, this limit concentration of 200 mg L^−1^ does not correspond to significant inhibition efficiency (about 62.5%); this is a critical limit for the acceleration in change for *R*_ct_ and *CPE*.

**Figure 8:**
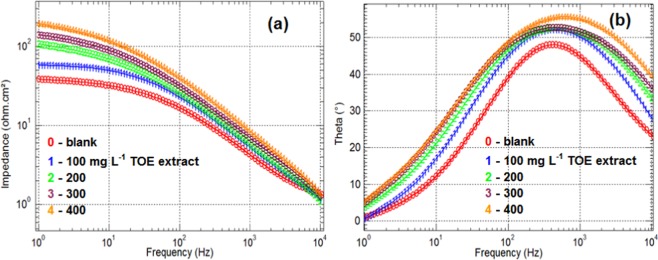
Bode plots – (**a**) modulus plots, and (**b**) phase angle plots for EIS tests.

The Bode plot is reported in Fig. 8. The impedance slightly decreases with increasing the frequency in the range 1–10–100 Hz before strongly dropping. At low frequency the impedance modulus is usually defined to evaluate the corrosion inhibition efficiency^59^. As expected, this inhibition efficiency increases with TOE dosage: there is a strong increase between 100 mg L^−1^ and 200 mg L^−1^, while at higher dosage the enhancement tends to stabilize. It is noteworthy that the range of frequency should be extended to lower frequencies (to 0.01–0.1 Hz) to reach the saturation and to get a better evaluation of corrosion inhibition efficiency^38^. The phase angle plots also confirm a slight increase of the angle and the width of the peak with increasing TOE dosage. This is associated to a more homogeneous coverage of the surface with TOE compounds. It is noteworthy that the peaks are relatively symmetrical; this means that there is only one time constant to be taken into account. Zhang *et al*.^23^ attributed the irregular shape of the angle plots for corrosion inhibition of aluminum by konjac glucomanan to the existence of two time constants associated with the formation of oxide film and double layer capacitance.

**Figure 9:**
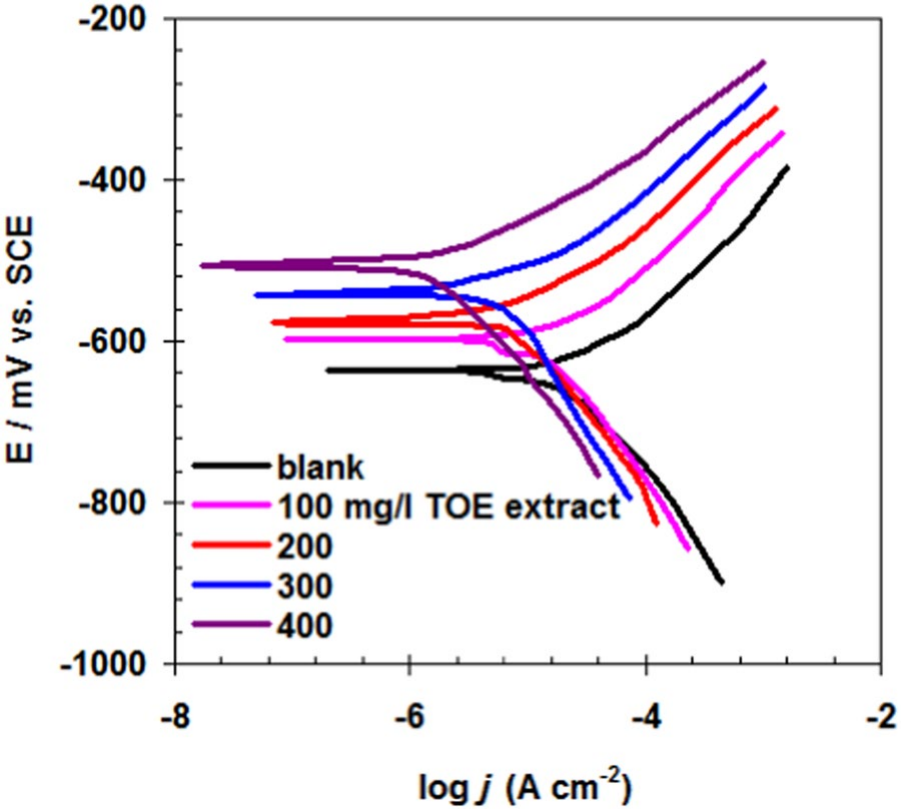
Tafel plot for carbon steel immersed in seawater - Comparison between raw seawater and seawater treated with increasing amounts of TOE (Experimental conditions: volume of solution: 100 ml, surface of steel sheet: 0.458 cm^2^, T: 298 K, scan rate: 1.25 mV s^−1^).

Poentiodyamic plarization is essential tool to understand the the role of TOE extract as corrosion inhibitor^60^. The plarization of carbon steel in raw seawater and seawater treated with increasing amounts of TOE extract was performed and the resulting Tafel plot is shown in Fig. 9. The polarization parameters as deduced from Fig. 9, i.e. corrosion potential (*E*_corr_), corrosion current density (*j*_corr_), cathodic and anodic Tafel slopes (*b*_c_, *b*_a_) are reported together with the inhibition efficiency (*η*_j_ %) in the Table 5.

**Table 5:** Polarization parameters and inhibition efficiency for carbon steel inhibition in seawater in the presence of increasing concentrations of TOE at 298 K.

TOE concentration (mg L^−1^)	*E*_corr._ mV (SCE)	*b*_a_ (mV dec^−1^)	-*b*_c_ (mV dec^−1^)	*j*_corr._ μA cm^−2^	*η*_j_%
0 - Blank	−632	68	187	15.80	—
100	−598	66	182	9.48	40.0
200	−576	59	181	6.42	59.3
300	−542	55	179	3.77	76.1
400	−507	53	176	1.85	88.2

The carbon steel material exhibited very low *j*_corr_ values in the presence of TOE extract (see Table 5). Thus clearly indicates that the ability of TOE extract to retard the corrosion reaction.

Moreover, the shifting of *E*_corr_ to a positive direction for all TOE extract concentrations confirms that this extract works predominantly as an anodic inhibitor^61^. Further, the *b*_c_ and *b*_a_ vary upon addition of TOE extract, which confirms that the TOE extract molecules are adsorbed on carbon steel surface including anodic and cathodic areas^62^.

When the TOE extract concentration is increased to 400 mg L^−1^, the *η*_j_% value reached 88.2% which implies that the TOE extract has a good anti-corrosion performance. Generally, from the mass loss, EIS and polarization studies, we confirm that the corrosion inhibition efficiency values from three methods are comparable.

### Surface observation and analysis

**Figure 10:**
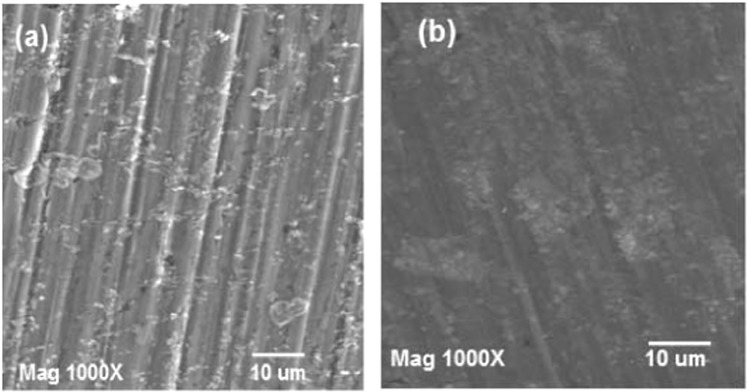
SEM micrographs for carbon steel sheets immersed in seawater (reference, **a**) and treated seawater (TOE, **b**) (Experimental conditions: dosage of TOE: 400 mg L^−1^, volume of solution: 100 ml, surface of steel sheet: 4.32 cm^2^, immersion time: 168 h, scale bar: 10 µm).

The surface morphology of carbon steel sheets submerged in seawater for 168 h with and without TOE extract is characterized using SEM. Figure 10a shows that seawater strongly corrodes the surface of carbon steel: pores appear on carbon steel sheet associated with an increase in surface roughness. On the opposite hand, the SEM of sample immersed in TOE-treated seawater shows a more regular and smooth surface (Fig. 10b): the TOE preserves carbon steel from corrosion damages. This is directly associated to the sorption of TOE compounds at carbon steel surface. It is noteworthy that the surface of the material exposed to TOE-seawater is homogenous: this means that the surface is totally covered by TOE extract (at least at 400 mg L^−1^ TOE dosage); indeed, a partial coverage of the surface would show inhomogeneity in the distribution of coating aggregates^63^.

**Figure 11:**
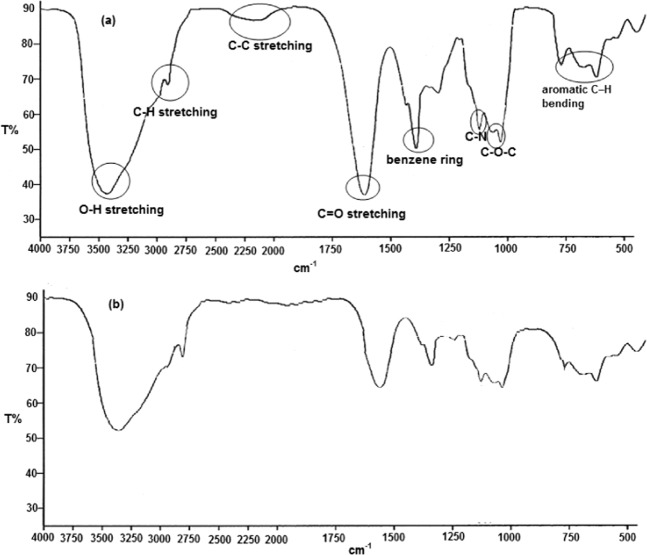
FTIR spectra of (**a**) pure TOE extract and (**b**) the film formed on the carbon steel surface during the immersion (168 h) in seawater solution containing 400 mg L^−1^ of TOE at 298 K.

FTIR spectra (see Fig. 11a) of TOE extract displays characterized peaks to O–H stretching (3390 cm^−1^), C-H stretching (2936.56 cm^−1^), C–C stretching (2150 cm^−1^), C=O stretching (1610 cm^−1^), benzene ring (1410 cm^−1^), C–N stretching (1130 cm^−1^), C–O–C stretching (1080 and 1110 cm^−1^), aromatic C–H bending (782 and 705 and 650 cm^−1^). This supports the data given by HPLC investigations (see Figs. 1 and 2).

To characterize the adsorption process of TOE extract molecules on the carbon steel surface, we examined FTIR spectra of the scratched film formed on the carbon steel surface after electrode immersion in seawater solution containing TOE extract (see Fig. 11b). Comparing the FTIR spectra in Fig. 10a,b confirms the adsorption process of TOE extract molecules on the carbon steel surface. The shifting in some peaks in Fig. 11b (such as O–H, C–H, C=O and benzene ring) comparing with Fig. 11a confirms that the adsorption process occurs via the functional groups present in TOE extract molecules.

### UV analysis of TOE-seawater

**Figure 12:**
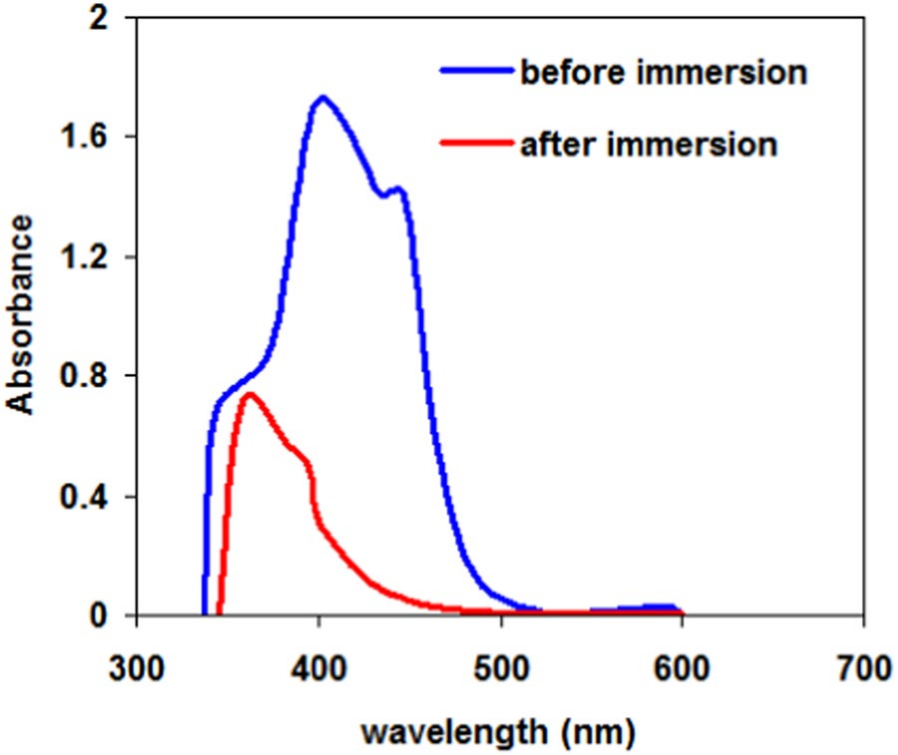
UV spectra of the TEO-seawater before and after immersion of the carbon steep sheet.

Figure 12 shows the UV spectra of the TOE-seawater before and after immersion of the carbon steel sample. The UV fingerprint strongly changes after contact with the metal sheet (and corrosion experiment). This is another clear evidence of the sorption mechanism. In the case of the use of *Citrus aurentifolia* extract for inhibiting corrosion of mild steel, Haldar *et al*.^55^ reported a shift in the band at 232.3 nm that was attributed to the n-π* transition for organic compounds; the intensity of this band was also strongly decreased. These two changes were assigned to the sorption of inhibitor molecules at the surface of steel sample. In the case of TOE, the changes are more significant. Indeed, the initial spectrum is characterized by three main peaks at around 340 nm, 420 nm and 480 nm. After corrosion test, the peak at 480 nm completely disappears, the peak at 420 nm is shifted toward 390–400 nm (with a strong decrease in intensity), while the peak at 340 nm maintains a similar intensity but with a shift toward higher wavelength (at 360 nm). This means that the different compounds present in the TOE extract are not similarly reacting with the metal surface during the corrosion process, in terms of sorption and/or degradation. In the case of P110SS steel corrosion, the natural dye berberine extract was used for inhibiting corrosion^57^; substantial changes were also observed on UV spectra before and after corrosion test. The peaks were affected both in terms of wavelength and intensity. These changes were attributed to π-π* and n-π* transitions in relation with change transfer mechanisms^57^. Ansari *et al*.^64^ performed DFT modeling for interpreting the mechanisms involved in corrosion inhibition in relation with donor-acceptor interactions (including π-electrons from inhibitor molecules and vacant d-orbitals of iron at steel surface).

### Inhibition mechanism

The different observations (SEM, FT-IR, sorption isotherm, UV and EIS) confirm that the sorption of organic compounds contained in the TOE constitutes the predominant mechanism of corrosion inhibition. The HPLC analysis isolated a series of carbohydrates^26^ that are probably involved in the mechanism of corrosion inhibition. However, some other polyphenols (not analyzed in this study) are also found in *Taraxacum officinale* leaves^26–28,33^, they may contribute to the binding on the surface of carbon steel surfaces and to the inhibition effect^36,65,66^.

These compounds contain O-bearing functional groups that have strong affinity for Fe at the surface of carbon steel: these compounds are bound by physisorption at steel surface partial sharing of O electrons and formation of double bonds, in addition to the binding of aromatic rings^12^. The adsorbed layer of organic compounds acts as a barrier between metal surface and corrosive solution^16,35,67,68^.

In seawater, most of the compounds present in TOE are neutral or cationic molecules. On the other side, carbon steel is supposed to be positively charged in seawater^16,56,69,70^. Therefore, the metal surface in seawater will first bound anionic compounds from seawater, which may occupy the surface; however, in a second step, the binding of these anionic charges may contribute to bind TOE cationic compounds through residual anionic charges.

Several authors modeled by quantum chemistry the mechanisms involved in the binding of model organic compounds at the surface of metal supports for preventing their corrosion. By HUMO/LUMO (electron donator/electron acceptor) modeling it is possible evaluating the optimized structure for their interaction with the support (involving the lower energy for electron withdrawing from highest occupied orbitals). It is generally accepted that a good organic-based inhibitor has the capacity to both donate free electrons to metal and accept free electrons from the metal^65^. The inhibitor effect is also controlled by the dipole moment of the inhibitor^65^: high dipole moments influence the dielectric characteristics of the electrical double layer, which, in turn, reduces the rate of electrochemical reactions^65^. The organic compounds contained in TOE have higher dipole moments than water; therefore these compounds easily replace water molecules at the surface of the metal sheet^61^. Ansari *et al*.^64^ commented that the inhibition is associated with the sorption of inhibitor molecules through:

(a) chemical mechanism (donor-acceptor interactions between free electron pairs of heteroatoms and π-electrons of multiple bonds and vacant d-orbitals of iron, (b) physical adsorption between charged metal surface and charged inhibitor molecules, and (c) the extra negative charge transfer from d-orbital of iron to vacant π* orbital of inhibitor molecules (retrodonation mechanism).

The sorption of TOE compounds at the surface of carbon steel sheets (through physisorption) protects the surface of the metal from corrosive solution. The protective layer affects the dielectric constant of the system with control on the electrochemical reactions.

## Conclusions

*Taraxacum officinale* extract (aqueous/ethanol solution) reveals an efficient new inhibitor for carbon steel in aggressive media such as seawater. The inhibition mechanism consists of the physisorption of the compounds contained in TOE. These compounds cover the surface of carbon steel sheets and the layer is preventing the corrosive attack of seawater to carbon steel sites. This protective layer and the adsorption of TOE compounds explain the inhibition effect.

The study of the effect of TOE concentration and temperature allows analyzing the thermodynamic parameters and confirming the protective effect of TOE layer. The system can be electrochemically schematized with a simple model involving the resistance of the aqueous solution, the capacitance and resistance of the electrical double layer. Nyquist and Bode plots confirm the improvement of inhibition effect with increasing TOE concentration: the charge transfer resistance strongly increases while the constant phase element (capacitance of the electrical double layer) drastically decreases. Moreover, the polarization data confirm that the TOE works predominantly as an anodic inhibitor.

## References

[1] Morales-Gil P (2015). Corrosion inhibition of carbon-steel with 2-mercaptobenzimidazole in hydrochloric acid. Corros. Sci..

[2] Hanza, A. P., Naderi, R., Kowsari, E. & Sayebani, M. Corrosion behavior of mild steel in H_2_SO_4_ solution with 1, 4-di 1′-methylene-3′-methyl imidazolium bromide -benzene as an ionic liquid. *Corros. Sci.* **107**, 96–106 (2016).

[3] Barmatov E, Hughes T, Eskin D (2016). Effect of surface roughness on corrosion behaviour of low carbon steel in inhibited 4 M hydrochloric acid under laminar and turbulent flow conditions. Corros. Sci..

[4] Deyab MAM (2015). Corrosion inhibition and adsorption behavior of sodium lauryl ether sulfate on L80 carbon steel in acetic acid solution and its synergism with ethanol. J. Surfactants Deterg..

[5] Chidiebere MA, Oguzie EE, Liu L, Li Y, Wang F (2015). Ascorbic acid as corrosion inhibitor for Q235 mild steel in acidic environments. J. Ind. Eng. Chem..

[6] Abdallah M (2017). Corrosion inhibition of stainless steel type 316 L in 1.0 M HCl solution using 1,3-thiazolidin-5-one derivatives. Int. J. Electrochem. Sci..

[7] Bentiss F, Lagrenee M, Traisnel M, Hornez JC (1999). The corrosion inhibition of mild steel in acidic media by a new triazole derivative. Corros. Sci..

[8] Shabani-Nooshabadi M, Ghandchi MS (2015). *Santolina chamaecyparissus* extract as a natural source inhibitor for 304 stainless steel corrosion in 3.5% NaCl. J. Ind. Eng. Chem..

[9] Deyab MA (2015). Egyptian licorice extract as a green corrosion inhibitor for copper in hydrochloric acid solution. J. Ind. Eng. Chem..

[10] Abdallah M, Altass HM, Al Jahdaly BA, Salem MM (2018). Some natural aqueous extracts of plants as green inhibitor for carbon steel corrosion in 0.5 M sulfuric acid. Green Chem. Lett. Rev..

[11] Ajeigbe SO, Aziz M, Basar N (2018). Adsorption and thermodynamic characteristics of phenylpropanoids of *Alpinia galanga* as corrosion inhibitors on mild steel. Adv. Sci. Lett..

[12] El Hamdani N, Fdil R, Tourabi M, Jama C, Bentiss F (2015). Alkaloids extract of *Retama monosperma (L.) Boiss*. seeds used as novel eco-friendly inhibitor for carbon steel corrosion in 1 M HCl solution: Electrochemical and surface studies. Appl. Surf. Sci..

[13] Marciales A, Haile T, Ahvazi B, Ngo TD, Wolodko J (2018). Performance of green corrosion inhibitors from biomass in acidic media. Corros. Rev..

[14] Pramudita, M., Sukirno, Nasikin, M. Influence of tannin content in *Terminalia catappa* leaves extracts resulted from maceration extraction on decreasing corrosion rate for mild steel in 1M H2SO4, in: Evelyn (Ed.) 2nd International Conference on Oleo and Petrochemical Engineering (2018).

[15] Deyab MA (2016). Corrosion inhibition of aluminum in biodiesel by ethanol extracts of *Rosemary leaves*. J. Taiwan Inst. Chem. Eng..

[16] Banerjee S, Srivastava V, Singh MM (2012). Chemically modified natural polysaccharide as green corrosion inhibitor for mild steel in acidic medium. Corros. Sci..

[17] Dob K, Zouaoui E, Zouied D (2018). Corrosion inhibition of curcuma and saffron on A106 Gr B carbon steel in 3% NaCl medium. Anti-Corros. Methods Mater..

[18] Fidrusli, A., Suryanto, Mahmood, M. Iop, Ginger extract as green corrosion inhibitor of mild steel in hydrochloric acid solution, In: *International Conference on Advances in Manufacturing and Materials Engineering* (2018).

[19] Komalasari, S. P., Utami, M. I., Fermi, Y. Aziz, R. S. Irianti, Corrosion control of carbon steel using inhibitor of banana peel extract in acid diluted solutions, In: Evelyn (Ed.) *2nd International Conference on Oleo and Petrochemical Engineering* (2018).

[20] Oulabbas, A., Abderrahmane, S. Natural extract of Opuntia ficus indica as green inhibitor for corrosion of XC52 steel in 1 M H_3_PO_4_, *Mater. Res. Express*, **6** (2019).

[21] Deyab MA (2016). Inhibition activity of seaweed extract for mild carbon steel corrosion in saline formation water. Desalination.

[22] Rodrigues LS, do Valle AF, D’Elia E (2018). Biomass of microalgae *Spirulina maxima* as a crrosion inhibitor for 1020 carbon steel in acidic solution. Int. J. Electrochem. Sci..

[23] Zhang KG (2018). Inhibitory effect of konjac glucomanan on pitting corrosion of AA5052 aluminium alloy in NaCl solution. J. Colloid Interface Sci..

[24] Duke, J. *Handbook of Medicinal Herbs*, 2nd Ed. ed., CRC Press, Boca Raton, FL, USA, 896 pp (2002).

[25] Lis B, Olas B (2019). Pro-health activity of dandelion (*Taraxacum officinale* L.) and its food products - history and present. J. Funct. Foods.

[26] Yarnell E, Abascal K (2009). Dandelion (*Taraxacum officinale* and *T mongolicum*). Integr. Med..

[27] Biel W, Jaroszewska A, Lyson E, Telesinski A (2017). The chemical composition and antioxidant properties of common dandelion leaves compared with sea buckthorn. Can. J. Plant. Sci..

[28] Escudero NL, De Arellano ML, Fernández S, Albarracín G, Mucciarelli S (2003). Taraxacum officinale as a food source. Plant Foods Hum. Nutr..

[29] Kenny O (2014). Investigating the potential of under-utilised plants from the *Asteraceae* family as a source of natural antimicrobial and antioxidant extracts. Food Chem..

[30] ASTM, Standard practice for preparing, cleaning and evaluating corrosion test specimens, in: G1, ASTM, West Conshhohocken, PA, USA (1999).

[31] John S, Jeevana R, Aravindakshan KK, Joseph A (2017). Corrosion inhibition of mild steel by N(4)-substituted thiosemicarbazone in hydrochloric acid media, Egypt. J. Pet..

[32] Deyab MA (2016). Decyl glucoside as a corrosion inhibitor for Magnesium–air battery. Journal of Power Sources.

[33] Jung Y (2011). Characterization of dandelion species using H-1 NMR- and GC-MS-based metabolite profiling. Analyst.

[34] Chellouli M (2016). Corrosion inhibition of iron in acidic solution by a green formulation derived from *Nigella sativa* L. Electrochim. Acta.

[35] Deng S, Li X (2012). Inhibition by *Ginkgo* leaves extract of the corrosion of steel in HCl and H_2_SO_4_ solutions. Corros. Sci..

[36] Soltani N (2014). *Silybum marianum* extract as a natural source inhibitor for 304 stainless steel corrosion in 1.0 M HCl. J. Ind. Eng. Chem..

[37] Prabakaran M (2017). *Aster koraiensis* as nontoxic corrosion inhibitor for mild steel in sulfuric acid. J. Ind. Eng. Chem..

[38] Rabizadeh T, Asl SK (2019). Casein as a natural protein to inhibit the corrosion of mild steel in HCl solution. J. Mol. Liq..

[39] Umoren SA, Solomon MM, Obot IB, Suleiman RK (2018). Comparative studies on the corrosion inhibition efficacy of ethanolic extracts of date palm leaves and seeds on carbon steel corrosion in 15% HCl solution. J. Adhes. Sci. Technol..

[40] Samontha A, Lugsanangarm K (2019). Corrosion inhibition and adsorption mechanism of eugenol on copper in HCl medium. Prot. Met. Phys. Chem..

[41] Deyab MA (2016). Electrochemical investigations on pitting corrosion inhibition of mild steel by provitamin B5 in circulating cooling water. Electrochimica Acta.

[42] Deyab MA (2016). The inhibition activity of butylated hydroxytoluene towards corrosion of carbon steel in biodiesel blend B20. J. Taiwan Inst. Chem. Eng..

[43] Srivastava M, Tiwari P, Srivastava SK, Prakash R, Ji G (2017). Electrochemical investigation of Irbesartan drug molecules as an inhibitor of mild steel corrosion in 1 M HCl and 0.5 M H_2_SO_4_ solutions. J. Mol. Liq..

[44] Obot IB, Umoren SA, Gasem ZM, Suleiman R, Ali BE (2015). Theoretical prediction and electrochemical evaluation of vinylimidazole and allylimidazole as corrosion inhibitors for mild steel in 1M HCl. J. Ind. Eng. Chem..

[45] Li X, Deng S, Fu H (2012). Inhibition of the corrosion of steel in HCl, H_2_SO_4_ solutions by bamboo leaf extract. Corros. Sci..

[46] Zheng XW, Gong M, Li Q (2017). Corrosion inhibition of mild steel in sulfuric acid solution by *Houttuynia cordata* extract. Int. J. Electrochem. Sci..

[47] Sharma, V., *et al* Use of *Sapindus (reetha)* as corrosion inhibitor of aluminium in acidic medium, *Mater. Res. Express*, **5** (2018).

[48] Cordeiro RFB, Belati AJS, Perrone D, D’Elia E (2018). Coffee husk as corrosion inhibitor for mild steel in HCl media. Int. J. Electrochem. Sci..

[49] Deyab MA (2015). Hydroxyethyl cellulose as efficient organic inhibitor of zinc-carbon battery corrosion in ammonium chloride solution: Electrochemical and surface morphology studies. J. Power Sources.

[50] Shimizu K, Lasia A, Boily J-F (2012). Electrochemical impedance study of the hematite/water interface. Langmuir.

[51] Wang C (2017). Inhibition of zinc corrosion by fucoidan in natural sea water. Acta Metall. Sinica - English Lett..

[52] Deyab MA (2015). Effect of carbon nano-tubes on the corrosion resistance of alkyd coating immersed in sodium chloride solution. Prog. Org. Coat..

[53] Ouici HB (2013). The effect of some triazole derivatives as inhibitors for the corrosion of mild steel in 5% hydrochloric acid. Res. Chem. Intermed..

[54] Njoku DI, Li Y, Lgaz H, Oguzie EE (2018). Dispersive adsorption of *Xylopia aethiopica* constituents on carbon steel in acid-chloride medium: A combined experimental and theoretical approach. J. Mol. Liq..

[55] Haldhar R, Prasad D, Bhardwaj N (2019). Extraction and experimental studies of *Citrus aurantifolia* as an economical and green corrosion inhibitor for mild steel in acidic media. J. Adhes. Sci. Technol..

[56] Li Y, Zhao P, Liang Q, Hou BR (2005). Berberine as a natural source inhibitor for mild steel in 1 M H_2_SO_4_. Appl. Surf. Sci..

[57] Li N (2019). The extraction of a natural dye berberine and evaluation of its corrosion inhibition properties for P110SS steel. Int. J. Electrochem. Sci..

[58] Tezeghdenti M, Etteyeb N, Dhouibi L, Kanoun O, Al-Hamri A (2017). Natural products as a source of environmentally friendly corrosion inhibitors of mild steel in dilute sulphuric acid: experimental and computational studies. Prot. Met. Phys. Chem..

[59] Recloux I (2018). Stability of benzotriazole-based films against AA2024 aluminium alloy corrosion process in neutral chloride electrolyte. J. Alloys Compd..

[60] Deyab MA (2016). Experimental evaluation of new inorganic phosphites as corrosion inhibitors for carbon steel in saline water from oil source wells. Desalination.

[61] Deyab MA, Zaky MT, Nessim MI (2017). Inhibition of acid corrosion of carbon steel using four imidazolium tetrafluoroborates *ionic liquids*. Journal of Molecular Liquids.

[62] Deyab MA (2016). NaNi(H_2_PO_3_)_3_.H_2_O as a novel corrosion inhibitor for X70-steel in saline produced water. Journal of Molecular Liquids.

[63] Kaskah SE, Ehrenhaft G, Gollnick J, Fischer CB (2019). Concentration and coating time effects of N-acyl sarcosine derivatives for corrosion protection of low-carbon steel CR4 in salt water - defining the window of application. Corros. Eng. Sci. Technol..

[64] Ansari KR, Quraishi MA, Singh A (2015). Corrosion inhibition of mild steel in hydrochloric acid by some pyridine derivatives: An experimental and quantum chemical study. J. Ind. Eng. Chem..

[65] Anupama KK, Ramya K, Shainy KM, Joseph A (2015). Adsorption and electrochemical studies of *Pimenta dioica* leaf extracts as corrosion inhibitor for mild steel in hydrochloric acid. Mater. Chem. Phys..

[66] Singh A (2015). *Gingko biloba* fruit extract as an eco-friendly corrosion inhibitor for J55 steel in CO_2_ saturated 3.5% NaCl solution. J. Ind. Eng. Chem..

[67] Abd El-Rehim, S. S., Hassan, H. H., Deyab, M. A. M. & Abd El, A. Moneim, Experimental and theoretical ivestigations of adsorption and inhibitive properties of Tween 80 on corrosion of aluminum alloy (A5754) in alkaline media, Z. *Phys. Chem. - Int. J. Res. Phys. Chem Chem. Phys.* **230**, 67–78 (2016).

[68] Deyab MA, Essehli R, El Bali B (2015). Performance evaluation of phosphite NaCo(H_2_PO_3)_)_3_.H_2_O as a corrosion inhibitor for aluminum in engine coolant solutions. RSC Adv..

[69] Bentiss F, Traisnel M, Lagrenee M (2000). The substituted 1,3,4-oxadiazoles: a new class of corrosion inhibitors of mild steel in acidic media. Corros. Sci..

[70] Obot IB, Obi-Egbedi NO, Odozi NW (2010). Acenaphtho 1, 2-b quinoxaline as a novel corrosion inhibitor for mild steel in 0.5 M H_2_SO_4_. Corros. Sci..

